# Calcioferrite with composition (Ca_3.94_Sr_0.06_)Mg_1.01_(Fe_2.93_Al_1.07_)(PO_4_)_6_(OH)_4_·12H_2_O

**DOI:** 10.1107/S1600536814004061

**Published:** 2014-02-28

**Authors:** Barbara Lafuente, Robert T. Downs, Hexiong Yang, Robert A. Jenkins

**Affiliations:** a Department of Geosciences, University of Arizona, 1040 E. 4th Street, Tucson, AZ 85721-0077, USA

## Abstract

Calcioferrite, ideally Ca_4_MgFe^3+^_4_(PO_4_)_6_(OH)_4_·12H_2_O (tetra­calcium magnesium tetrairon(III) hexakis-phosphate tetra­hydroxide dodeca­hydrate), is a member of the calcioferrite group of hydrated calcium phosphate minerals with the general formula Ca_4_ *AB*_4_(PO_4_)_6_(OH)_4_·12H_2_O, where *A* = Mg, Fe^2+^, Mn^2+^ and *B* = Al, Fe^3+^. Calcioferrite and the other three known members of the group, montgomeryite (*A* = Mg, *B* = Al), kingsmountite (*A* = Fe^2+^, *B* = Al), and zodacite (*A* = Mn^2+^, *B* = Fe^3+^), usually occur as very small crystals, making their structure refinements by conventional single-crystal X-ray diffraction challenging. This study presents the first structure determination of calcioferrite with composition (Ca_3.94_Sr_0.06_)Mg_1.01_(Fe_2.93_Al_1.07_)(PO_4_)_6_(OH)_4_·12H_2_O based on single-crystal X-ray diffraction data collected from a natural sample from the Moculta quarry in Angaston, Australia. Calcioferrite is isostructural with montgomeryite, the only member of the group with a reported structure. The calcioferrite structure is characterized by (Fe/Al)O_6_ octa­hedra (site symmetries 2 and -1) sharing corners (OH) to form chains running parallel to [101]. These chains are linked together by PO_4_ tetra­hedra (site symmetries 2 and 1), forming [(Fe/Al)_3_(PO_4_)_3_(OH)_2_] layers stacking along [010], which are connected by (Ca/Sr)^2+^ cations (site symmetry 2) and Mg^2+^ cations (site symmetry 2; half-occupation). Hydrogen-bonding inter­actions involving the water mol­ecules (one of which is equally disordered over two positions) and OH function are also present between these layers. The relatively weaker bonds between the layers account for the cleavage of the mineral parallel to (010).

## Related literature   

For background references to calcioferrite, see: Blum (1858 ▶); Palache *et al.* (1951 ▶); Henderson & Peisley (1985 ▶). For discussions on minerals isostructural with calcioferrite, see: Larsen (1940 ▶); Moore & Araki (1974 ▶); Fanfani *et al.* (1976 ▶); Dunn *et al.* (1979 ▶, 1983 ▶, 1988 ▶). For information on phosphate minerals, see: Mead & Mrose (1968 ▶); Huminicki & Hawthorne (2002 ▶). For details of rigid-body thermal motion of atoms in crystals, see: Downs (2000 ▶).

## Experimental   

### 

#### Crystal data  

(Ca_3.94_Sr_0.06_)Mg_1.01_(Fe_2.93_Al_1.07_)(PO_4_)_6_(OH)_4_·12H_2_O
*M* *_r_* = 1234.28
Monoclinic, 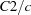

*a* = 10.1936 (8) Å
*b* = 24.1959 (18) Å
*c* = 6.3218 (4) Å
β = 91.161 (4)°
*V* = 1558.9 (2) Å^3^
*Z* = 2
Mo *K*α radiation
μ = 2.60 mm^−1^
*T* = 293 K
0.09 × 0.08 × 0.05 mm

#### Data collection  

Bruker APEXII CCD area-detector diffractometer
Absorption correction: multi-scan (*SADABS*; Bruker, 2004 ▶) *T*_min_ = 0.800, *T*_max_ = 0.881
10490 measured reflections
2348 independent reflections
1712 reflections with *I* > 2σ(*I*)
*R*_int_ = 0.049

#### Refinement  

*R*[*F* ^2^ > 2σ(*F* ^2^)] = 0.039
*wR*(*F* ^2^) = 0.094
*S* = 1.01
2348 reflections
160 parameters
4 restraints
All H-atom parameters refined
Δρ_max_ = 0.57 e Å^−3^
Δρ_min_ = −0.60 e Å^−3^

### 

Data collection: *APEX2* (Bruker, 2004 ▶); cell refinement: *SAINT* (Bruker, 2004 ▶); data reduction: *SAINT*; program(s) used to solve structure: *SHELXS97* (Sheldrick, 2008 ▶); program(s) used to refine structure: *SHELXL97* (Sheldrick, 2008 ▶); molecular graphics: *XtalDraw* (Downs & Hall-Wallace, 2003 ▶); software used to prepare material for publication: *publCIF* (Westrip, 2010 ▶).

## Supplementary Material

Crystal structure: contains datablock(s) I. DOI: 10.1107/S1600536814004061/wm5004sup1.cif

Structure factors: contains datablock(s) I. DOI: 10.1107/S1600536814004061/wm5004Isup2.hkl

CCDC reference: 988095

Additional supporting information:  crystallographic information; 3D view; checkCIF report

## Figures and Tables

**Table 1:** Hydrogen-bond geometry (Å, °)

*D*—H⋯*A*	*D*—H	H⋯*A*	*D*⋯*A*	*D*—H⋯*A*
O*W*1—H11⋯O3^i^	0.72 (3)	2.40 (4)	2.994 (4)	142 (5)
O*W*1—H11⋯O6^i^	0.72 (3)	2.41 (3)	3.079 (4)	155 (5)
O*W*1—H12⋯O*H*	0.74 (3)	2.45 (3)	3.145 (4)	159 (4)
O*W*1—H12⋯O2	0.74 (3)	2.75 (4)	3.179 (4)	120 (4)
O*W*2—H21⋯O5	1.00 (4)	1.61 (4)	2.606 (3)	174 (4)
O*W*2—H22⋯O*W*3*B* ^i^	0.86 (4)	2.02 (4)	2.841 (9)	160 (4)
O*W*2—H22⋯O*W*3*A* ^i^	0.86 (4)	2.27 (4)	3.033 (10)	148 (4)
O*W*2—H22⋯O6^ii^	0.86 (4)	2.57 (4)	2.973 (4)	109 (3)
O*H*—H1⋯O*W*1^i^	0.69 (4)	2.22 (4)	2.891 (4)	165 (5)

Symmetry codes: (i) 
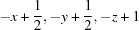
; (ii) 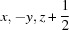.

**Fig. 1.**
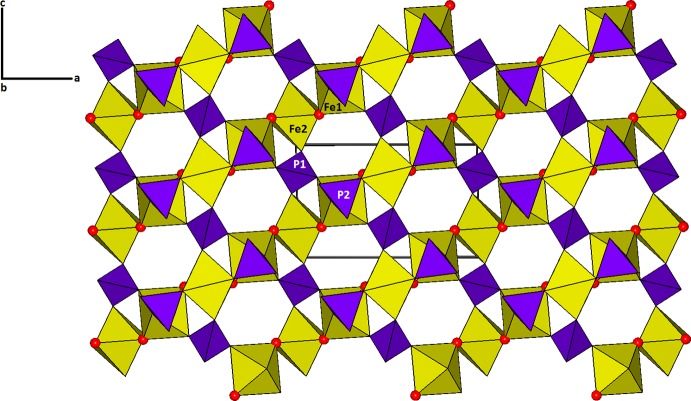
A slice of the calcioferrite structure, showing the [(Fe/Al)3(PO4)3(OH)2] layer. Yellow octahedra, purple tetrahedra and red spheres represent (Fe/Al)O6, PO4 and OH groups, respectively.

**Fig. 2.**
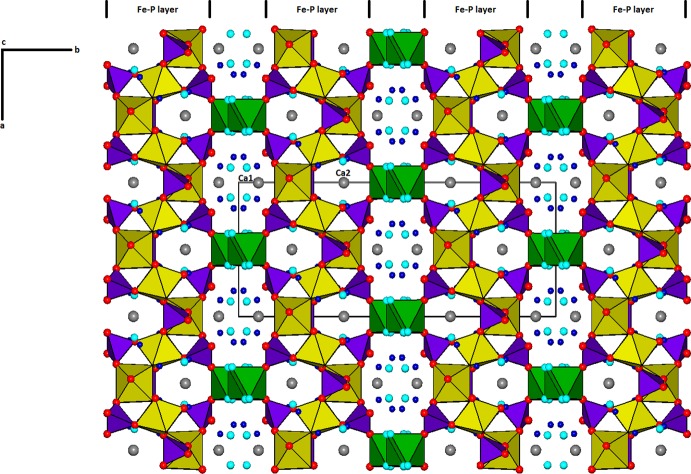
The [(Fe/Al)3(PO4)3(OH)2] layers are stacked along [010]. They are connected by H2O and cations Ca2+ and Mg2+. Yellow and green octahedra represent (Fe/Al)O6 and MgO6 groups, respectively. Purple tetrahedra represent PO4 groups. Grey, aquamarine, blue and red spheres represent Ca2+ cations, H2O molecules, H atoms and O atoms, respectively.

**Fig. 3.**
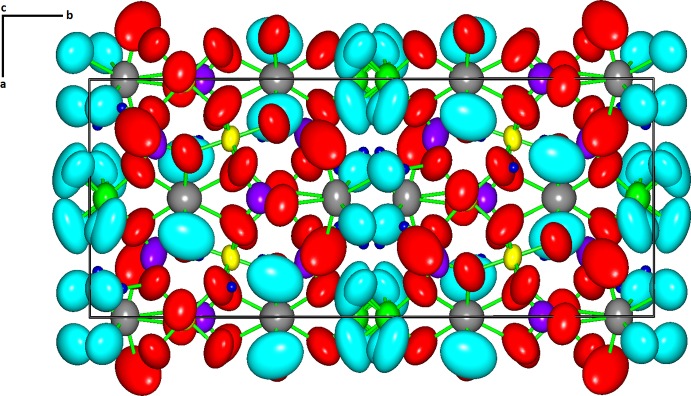
The crystal structure of calcioferrite showing atoms with anisotropic displacement ellipsoids at the 99% probability level. Yellow, purple, green and grey ellipsoids represent (Fe/Al), P, Mg and Ca sites, respectively. Red and aquamarine ellipsoids represent O atoms and H2O groups, respectively. Small blue spheres represent H atoms.

**Fig. 4.**
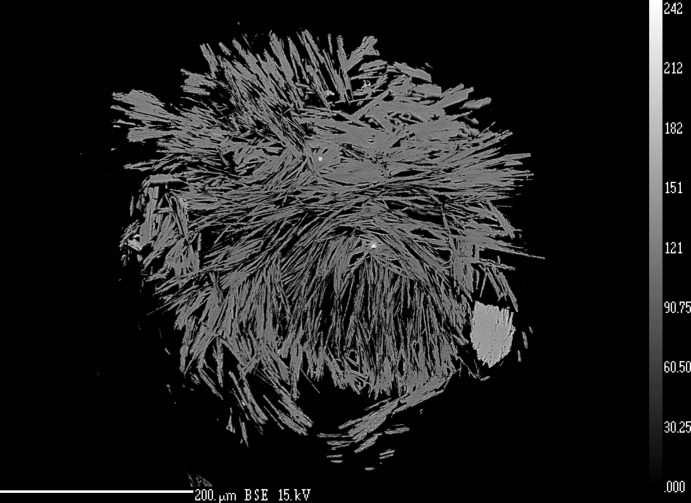
A backscattered electron image of calcioferrite crystals.

## supplementary crystallographic information

### 1. Comment

Calcioferrite was originally described by Blum (1858) from a sample found in Battenberg (Rhenish, Bavaria) with the chemical composition (wt.%): P_2_O_5_ 34.01, Fe_2_O_3_ 24.34, Al_2_O_3_ 2.90, CaO 14.81, MgO 2.65, and H_2_O 20.56 (total = 99.27). Larsen (1940) reported montgomeryite with an ideal chemical formula Ca_4_Al_5_(PO_4_)_6_(OH)_5_.11H_2_O without recognizing its relationship to calcioferrite. Palache *et al.* (1951), on the basis of the chemistry given by Blum (1858), proposed the chemical formula Ca_3_Fe_3_(PO_4_)_4_(OH)_3_.8H_2_O for calcioferrite. By comparing chemical compositions and X-ray powder diffraction profiles between calcioferrite and montgomeryite, Mead & Mrose (1968) suggested that these two minerals are isostructural. Moore & Araki (1974) first solved the structure of montgomeryite in space group *C*2/*c* and revised its chemical formula to Ca_4_MgAl_4_(PO_4_)_6_(OH)_4_.12H_2_O. Nevertheless, Fanfani *et al.* (1976) observed the presence of some weak reflections that violate the *C*2/*c* space group symmetry for montgomeryite, leading them to propose *C*2 as the actual space group for this mineral. Dunn *et al.* (1983) studied red montgomeryite from the Tip Top Pegmatite and also concluded that calcioferrite is the Fe^3+^ analog of montgomeryite based on the similarity between their X-ray powder diffraction patterns. Consequently, they modified the ideal chemical formula of calcioferrite to its present form, Ca_4_MgFe^3+^_4_(PO_4_)_6_(OH)_4_.12H_2_O.

A second locality for calcioferrite was reported by Henderson & Peisley (1985) at the Moculta quarry in Angaston, South Australia, associated with apatite, jarosite, cacoxenite and altered pyrite, the latter probably being the source of Fe^3+^. The chemistry and X-ray power data of calcioferrite from this locality are consistent with the previous observations that calcioferrite is isotypic with montgomeryite. However, the structure of calcioferrite has remained undetermined because of its small crystal size and generally poor crystallinity. In the course of identifying minerals for the RRUFF Project (http://rruff.info), we were able to isolate a single crystal of calcioferrite and determine its structure by means of single-crystal X-ray diffraction, demonstrating that its space group is *C*2/*c*.

The general composition of the calcioferrite group minerals can be expressed as Ca_4_*AB*_4_(PO_4_)_6_(OH)_4_.12H_2_O with *A* = Mg, Fe^2+^, Mn^2+^ and *B* = Al, Fe^3+^. In addition to calcioferrite, there are three other known members in the group, including montgomeryite (*A* = Mg, *B* = Al) (Moore & Araki, 1974; Fanfani *et al.*, 1976), kingsmountite (*A* = Fe^2+^, *B* = Al) (Dunn *et al.*, 1979), and zodacite (*A* = Mn^2+^, *B* = Fe^3+^) (Dunn *et al.*, 1988). The structure of calcioferrite contains seven non-hydrogen cation sites, two for Ca [((Cal/Sr1); site symmetry 2; occupancy ratio Ca:Sr =0.97:0.03) and Ca2 (site symmetry 2)], two for Fe [((Fe1/Al1); site symmetry 1; occupancy ratio Fe:Al = 0.651:0.349) and (Fe2/Al2; site symmetry 2; occupancy ratio Fe:Al = 0.814:0.186)], one for Mg [site symmetry 2; half-occupation], and two for P [(P1; site symmetry 2) and P2 (site symmetry 1)]. The chains of corner-sharing (Fe/Al)O_6_ o­cta­hedra (parallel to [101]) are linked together by PO_4_ tetra­hedra to form [(Fe/Al)_3_(PO_4_)_3_(OH)_2_] layers stacking along [010] (Figs. 1, 2). The configuration of such layers has been observed in many others (Fe/Al)^3+^ phosphates (Huminicki & Hawthorne, 2002). The [(Fe/Al)_3_(PO_4_)_3_(OH)_2_] layers are connected by Ca^2+^ cations (coordination numbers of eight) and Mg^2+^ cations (coordination number of six). The relatively weaker bonds between the layers account for the cleavage of the mineral parallel to (010).

The (Fe/Al)O_6_ o­cta­hedral chains in calcioferrite have a repeat of ~7.1 Å, similar to those examined by Huminicki & Hawthorne (2002). Between the two distinct *B* sites, the *B*1 site is strongly preferred by Al. The average (Fe/Al)1—O distance is 1.962 Å, which is evidently shorter than the average (Fe/Al)2—O distance (1.997 Å). The analysis of the anisotropic displacement parameters of atoms indicates that PO_4_ tetra­hedra behave as rigid bodies, as should be expected for such strongly bonded tetra­hedral groups (Downs, 2000). Both (Ca/Sr)1 and Ca2 are eight-coordinated, with the former by (4 O + 4 H_2_O) and the latter by (6 O + 2 H_2_O). The (Ca/Sr)1O_8_ polyhedra are situated between the [(Fe/Al)_3_(PO_4_)_3_(OH)_2_] layers, whereas the Ca2O_8_ polyhedra are located within the layers (Fig. 2). Hydrogen-bonding inter­actions involving the water molecules and OH- function are also present between these layers (Table 1).

As observed for the (Ca/Sr)1O_8_ polyhedra, the MgO_6_ o­cta­hedra are also located between the [(Fe/Al)_3_(PO_4_)_3_(OH)_2_] layers (Fig. 2). The Mg-site is randomly half-occupied with an average Mg—O bond length of 1.988 Å. The water O atom OW3 appears to be split between two positions (OW3A and OW3B), representing the two sets of water molecules correlated to the occupancy of the Mg-site (Fig. 3). The displacement parameters for OW3A are significantly larger and elongated than those of OW3B, suggesting that OW3A correlates with the vacancy and therefore is in a "softer" potential well. Inter­atomic distances between Mg—OW3B are more similar to each other (2.145 (8) Å and 2.200 (9) Å) while those between Mg—OW3A are more dissimilar to each other (2.535 (11) Å and 2.117 (10) Å). This is consistent with our suggestion, based on displacement parameters, that OW3A is not bonded to Mg.

### 1.1. Experimental

The calcioferrite specimen used in this study is from Moculta quarry, Angaston, Australia, and is in the collection of the RRUFF project (deposition R120092: http://rruff.info/R120092). Its chemical composition was determined with a CAMECA SX100 electron microprobe at the conditions of 15kV, 1nA and a beam size of 5µm. These conditions were optimized to minimize sample damage by the electron beam due to the small size of the sample (Fig. 4) and its high hydration. Ten analysis points yielded an average composition (wt. %): CaO 17.40 (41), SrO 0.57 (21), MgO 3.24 (16), Fe_2_O_3_ 18.51(1.44), Al_2_O_3_ 4.29 (86) and P_2_O_5_ 34.97 (86), with H_2_O 21.02 calculated by difference. Due to the significant dehydration of the sample during the electron microprobe analysis, this composition may not be very accurate and was used only for the estimation of cation ratios. By assuming six P cations per formula, the relative ratio of (Ca, Sr):Mg:(Fe, Al):P is 3.85:0.98:3.85:6.00. The composition of the crystal is then (Ca_3.94_Sr_0.06_)_Σ__=4_Mg(Fe_2.93_Al_1.07_)_Σ__=4_(PO_4_)_6_(OH)_4_.12H_2_O as determined by the combination of the electron microprobe and the X-ray structural data.

### 1.2. Refinement

All non-hydrogen atoms were refined with anisotropic displacement parameters. Only H atoms bonded to OW1, OW2, and OH could be located from difference Fourier syntheses and their positions refined with a fixed isotropic displacement parameter (*U*_iso_ = 0.03). The H atoms bonded to the disordered OW3 atom could not be located and were excluded from refinement.

The occupancies of Al and Fe of the two *B* sites were refined with their ratio determined from the electron microprobe analysis. The small amount of Sr detected from the electron microprobe analysis was assigned into the Ca1 site, because this site is significantly larger than the Ca2 site. The maximum residual electron density in the difference Fourier map, 0.57 e/Å^3^, was located at (0, 0.0340, 0.25), 0.69 Å from Sr1 and the minimum, -0.60 e/Å^3^, at (0.8637, 0.3318, 0.0082), 1.31 Å from OW1.

### Crystal data

**Table:**

Ca_4_MgFe_4_(PO_4_)_6_(OH)_4_·12H_2_O	*F*(000) = 1243
*M**_r_* = 1234.28	*D*_x_ = 2.629 Mg m^−^^3^
Monoclinic, *C*2/*c*	Mo *K*α radiation, λ = 0.71073 Å
Hall symbol: -C 2yc	Cell parameters from 2019 reflections
*a* = 10.1936 (8) Å	θ = 2.2–29.9°
*b* = 24.1959 (18) Å	µ = 2.60 mm^−^^1^
*c* = 6.3218 (4) Å	*T* = 293 K
β = 91.161 (4)°	Plate, pale yellow
*V* = 1558.9 (2) Å^3^	0.09 × 0.08 × 0.05 mm
*Z* = 2	

### Data collection

**Table:**

Bruker APEXII CCD area-detector diffractometer	2348 independent reflections
Radiation source: fine-focus sealed tube	1712 reflections with *I* > 2σ(*I*)
Graphite monochromator	*R*_int_ = 0.049
φ and ω scan	θ_max_ = 30.5°, θ_min_ = 2.2°
Absorption correction: multi-scan (*SADABS*; Bruker, 2004)	*h* = −14→14
*T*_min_ = 0.800, *T*_max_ = 0.881	*k* = −34→34
10490 measured reflections	*l* = −8→8

### Refinement

**Table:**

Refinement on *F*^2^	Secondary atom site location: difference Fourier map
Least-squares matrix: full	Hydrogen site location: difference Fourier map
*R*[*F*^2^ > 2σ(*F*^2^)] = 0.039	All H-atom parameters refined
*wR*(*F*^2^) = 0.094	*w* = 1/[σ^2^(*F*_o_^2^) + (0.0509*P*)^2^] where *P* = (*F*_o_^2^ + 2*F*_c_^2^)/3
*S* = 1.01	(Δ/σ)_max_ = 0.002
2348 reflections	Δρ_max_ = 0.57 e Å^−^^3^
160 parameters	Δρ_min_ = −0.60 e Å^−^^3^
4 restraints	Extinction correction: *SHELXL97* (Sheldrick, 2008), Fc^*^=kFc[1+0.001xFc^2^λ^3^/sin(2θ)]^-1/4^
Primary atom site location: structure-invariant direct methods	Extinction coefficient: 0.0012 (3)

### Special details

**Table:**

Geometry. All e.s.d.'s (except the e.s.d. in the dihedral angle between two l.s. planes) are estimated using the full covariance matrix. The cell e.s.d.'s are taken into account individually in the estimation of e.s.d.'s in distances, angles and torsion angles; correlations between e.s.d.'s in cell parameters are only used when they are defined by crystal symmetry. An approximate (isotropic) treatment of cell e.s.d.'s is used for estimating e.s.d.'s involving l.s. planes.
Refinement. Refinement of *F*^2^ against ALL reflections. The weighted *R*-factor *wR* and goodness of fit *S* are based on *F*^2^, conventional *R*-factors *R* are based on *F*, with *F* set to zero for negative *F*^2^. The threshold expression of *F*^2^ > σ(*F*^2^) is used only for calculating *R*-factors(gt) *etc*. and is not relevant to the choice of reflections for refinement. *R*-factors based on *F*^2^ are statistically about twice as large as those based on *F*, and *R*- factors based on ALL data will be even larger.

### Fractional atomic coordinates and isotropic or equivalent isotropic displacement parameters (Å^2^)

**Table:**

	*x*	*y*	*z*	*U*_iso_*/*U*_eq_	Occ. (<1)
Ca1	0.0000	0.06262 (3)	0.2500	0.0138 (2)	0.9700 (1)
Sr1	0.0000	0.06262 (3)	0.2500	0.0138 (2)	0.0300 (1)
Ca2	0.0000	0.33191 (4)	0.2500	0.0155 (2)	
Mg	0.0000	0.47193 (12)	0.2500	0.0130 (6)	0.5050 (1)
Fe1	0.2500	0.2500	0.0000	0.0076 (2)	0.651 (3)
Al1	0.2500	0.2500	0.0000	0.0076 (2)	0.349 (3)
Fe2	0.0000	0.16865 (3)	−0.2500	0.00762 (17)	0.814 (3)
Al2	0.0000	0.16865 (3)	−0.2500	0.00762 (17)	0.186 (3)
P1	0.5000	0.30208 (5)	−0.2500	0.0115 (2)	
P2	0.26354 (8)	0.11351 (3)	0.96145 (13)	0.01383 (19)	
O1	0.6135 (2)	0.26286 (10)	0.7074 (4)	0.0198 (5)	
O2	0.4700 (2)	0.34019 (9)	0.5625 (3)	0.0164 (5)	
O3	0.3130 (2)	0.17285 (9)	0.0051 (4)	0.0189 (5)	
O4	0.3782 (2)	0.08573 (9)	0.8546 (4)	0.0201 (5)	
O5	0.1438 (2)	0.11435 (9)	0.8055 (4)	0.0192 (5)	
O6	0.2234 (3)	0.08585 (10)	0.1650 (4)	0.0284 (6)	
OH	0.3675 (2)	0.27149 (10)	0.2343 (4)	0.0173 (5)	
OW1	0.1598 (3)	0.32890 (13)	0.5225 (4)	0.0314 (7)	
OW2	0.1120 (2)	0.02555 (10)	0.5770 (4)	0.0229 (5)	
OW3A	0.1164 (9)	0.4665 (4)	0.6075 (13)	0.0281 (17)	0.50
OW3B	0.1193 (8)	0.4792 (3)	0.5320 (12)	0.0199 (15)	0.50
H11	0.178 (4)	0.3424 (18)	0.620 (6)	0.030*	
H12	0.213 (4)	0.3114 (17)	0.483 (7)	0.030*	
H21	0.123 (4)	0.0581 (17)	0.673 (6)	0.030*	
H22	0.191 (4)	0.0156 (17)	0.553 (6)	0.030*	
H1	0.372 (4)	0.2496 (18)	0.303 (7)	0.030*	

### Atomic displacement parameters (Å^2^)

**Table:**

	*U*^11^	*U*^22^	*U*^33^	*U*^12^	*U*^13^	*U*^23^
Ca1	0.0177 (4)	0.0103 (4)	0.0132 (4)	0.000	−0.0008 (3)	0.000
Sr1	0.0177 (4)	0.0103 (4)	0.0132 (4)	0.000	−0.0008 (3)	0.000
Ca2	0.0149 (4)	0.0155 (5)	0.0160 (5)	0.000	0.0010 (3)	0.000
Mg	0.0115 (14)	0.0084 (14)	0.0192 (16)	0.000	0.0000 (11)	0.000
Fe1	0.0081 (4)	0.0043 (3)	0.0104 (4)	−0.0008 (3)	0.0006 (2)	−0.0003 (3)
Al1	0.0081 (4)	0.0043 (3)	0.0104 (4)	−0.0008 (3)	0.0006 (2)	−0.0003 (3)
Fe2	0.0088 (3)	0.0055 (3)	0.0087 (3)	0.000	0.0011 (2)	0.000
Al2	0.0088 (3)	0.0055 (3)	0.0087 (3)	0.000	0.0011 (2)	0.000
P1	0.0129 (5)	0.0102 (5)	0.0113 (5)	0.000	0.0019 (4)	0.000
P2	0.0160 (4)	0.0093 (4)	0.0164 (4)	0.0018 (3)	0.0049 (3)	0.0028 (3)
O1	0.0223 (12)	0.0176 (12)	0.0198 (12)	0.0062 (9)	0.0083 (9)	0.0031 (9)
O2	0.0220 (12)	0.0147 (11)	0.0127 (11)	0.0020 (9)	0.0022 (8)	0.0001 (8)
O3	0.0204 (12)	0.0129 (11)	0.0233 (13)	0.0027 (9)	−0.0031 (9)	−0.0033 (9)
O4	0.0196 (12)	0.0152 (12)	0.0258 (13)	0.0056 (9)	0.0068 (9)	0.0019 (9)
O5	0.0160 (12)	0.0132 (12)	0.0282 (13)	0.0031 (9)	−0.0034 (9)	−0.0036 (10)
O6	0.0324 (14)	0.0273 (15)	0.0261 (14)	0.0048 (11)	0.0138 (11)	0.0118 (11)
OH	0.0197 (12)	0.0115 (12)	0.0206 (13)	−0.0040 (9)	−0.0034 (9)	0.0048 (9)
OW1	0.0299 (16)	0.0415 (19)	0.0226 (15)	−0.0034 (13)	−0.0055 (11)	0.0040 (13)
OW2	0.0221 (13)	0.0194 (13)	0.0271 (14)	−0.0027 (11)	−0.0022 (10)	−0.0048 (10)
OW3A	0.040 (4)	0.018 (4)	0.026 (5)	0.009 (3)	−0.006 (4)	−0.005 (3)
OW3B	0.018 (3)	0.015 (4)	0.026 (5)	0.005 (2)	−0.008 (3)	0.000 (3)

### Geometric parameters (Å, º)

**Table:**

Ca1—O6^i^	2.417 (2)	Mg—OW3B^viii^	2.200 (9)
Ca1—O6	2.417 (2)	Mg—OW3A^i^	2.535 (8)
Ca1—OW2^i^	2.507 (3)	Mg—OW3A	2.535 (8)
Ca1—OW2	2.507 (3)	Fe1—O1^iii^	1.956 (2)
Ca1—O2^ii^	2.648 (2)	Fe1—O1^x^	1.956 (2)
Ca1—O2^iii^	2.648 (2)	Fe1—OH^vii^	1.956 (2)
Ca1—OW2^iv^	2.665 (3)	Fe1—OH	1.956 (2)
Ca1—OW2^v^	2.665 (3)	Fe1—O3	1.974 (2)
Ca2—OW1^i^	2.348 (3)	Fe1—O3^vii^	1.974 (2)
Ca2—OW1	2.348 (3)	Fe2—OH^vii^	1.981 (2)
Ca2—O4^ii^	2.446 (2)	Fe2—OH^iii^	1.981 (2)
Ca2—O4^iii^	2.446 (2)	Fe2—O5^i^	1.995 (2)
Ca2—O3^vi^	2.524 (2)	Fe2—O5^xi^	1.995 (2)
Ca2—O3^vii^	2.524 (2)	Fe2—O2^vii^	2.016 (2)
Ca2—O1^ii^	2.585 (3)	Fe2—O2^iii^	2.016 (2)
Ca2—O1^iii^	2.585 (3)	P1—O1^x^	1.525 (2)
Mg—O4^iii^	1.990 (3)	P1—O1^xi^	1.525 (2)
Mg—O4^ii^	1.990 (3)	P1—O2^x^	1.528 (2)
Mg—OW3A^viii^	2.117 (10)	P1—O2^xi^	1.528 (2)
Mg—OW3A^ix^	2.117 (10)	P2—O6^xii^	1.514 (2)
Mg—OW3B^i^	2.145 (8)	P2—O4	1.519 (2)
Mg—OW3B	2.145 (8)	P2—O3^xii^	1.545 (2)
Mg—OW3B^ix^	2.200 (9)	P2—O5	1.553 (2)
			
O4^iii^—Mg—O4^ii^	90.93 (18)	O1^iii^—Fe1—OH^vii^	91.85 (10)
O4^iii^—Mg—OW3A^viii^	173.6 (2)	O1^x^—Fe1—OH^vii^	88.15 (10)
O4^ii^—Mg—OW3A^viii^	89.6 (2)	OH^vii^—Fe1—OH	180.00 (13)
OW3A^viii^—Mg—OW3A^ix^	90.5 (5)	O1^iii^—Fe1—O3	94.26 (10)
O4^iii^—Mg—OW3B^i^	89.3 (3)	O1^x^—Fe1—O3	85.74 (10)
O4^ii^—Mg—OW3B^i^	97.4 (2)	OH^vii^—Fe1—O3	87.43 (10)
OW3A^viii^—Mg—OW3B^i^	84.3 (4)	OH—Fe1—O3	92.57 (10)
OW3A^ix^—Mg—OW3B^i^	89.0 (2)	O3—Fe1—O3^vii^	180.0
OW3B^i^—Mg—OW3B	170.5 (5)	OH^vii^—Fe2—OH^iii^	86.06 (14)
O4^iii^—Mg—OW3B^ix^	79.2 (2)	OH^vii^—Fe2—O5^i^	171.18 (9)
O4^ii^—Mg—OW3B^ix^	160.24 (19)	OH^iii^—Fe2—O5^i^	88.56 (10)
OW3A^viii^—Mg—OW3B^ix^	102.1 (3)	O5^i^—Fe2—O5^xi^	97.60 (13)
OW3A^ix^—Mg—OW3B^ix^	15.01 (19)	OH^vii^—Fe2—O2^vii^	90.57 (9)
OW3B^i^—Mg—OW3B^ix^	99.5 (3)	OH^iii^—Fe2—O2^vii^	98.35 (9)
OW3B—Mg—OW3B^ix^	75.3 (3)	O5^xi^—Fe2—O2^vii^	88.68 (9)
OW3B^ix^—Mg—OW3B^viii^	115.1 (4)	O2^vii^—Fe2—O2^iii^	167.81 (13)
O4^iii^—Mg—OW3A^i^	88.6 (2)	O1^x^—P1—O1^xi^	103.03 (19)
O4^ii^—Mg—OW3A^i^	87.2 (2)	O1^x^—P1—O2^x^	112.29 (12)
OW3A^viii^—Mg—OW3A^i^	85.0 (3)	O1^xi^—P1—O2^x^	111.83 (13)
OW3A^ix^—Mg—OW3A^i^	99.2 (4)	O2^x^—P1—O2^xi^	105.75 (18)
OW3B^i^—Mg—OW3A^i^	10.2 (3)	O6^xii^—P2—O4	113.94 (14)
OW3B—Mg—OW3A^i^	173.1 (4)	O6^xii^—P2—O3^xii^	110.61 (14)
OW3B^ix^—Mg—OW3A^i^	109.38 (17)	O4—P2—O3^xii^	103.84 (13)
OW3B^viii^—Mg—OW3A^i^	74.0 (3)	O6^xii^—P2—O5	108.83 (14)
OW3A^i^—Mg—OW3A	174.1 (4)	O4—P2—O5	109.00 (13)
O1^iii^—Fe1—O1^x^	180.0	O3^xii^—P2—O5	110.54 (13)

Symmetry codes: (i) −*x*, *y*, −*z*+1/2; (ii) −*x*+1/2, −*y*+1/2, −*z*+1; (iii) *x*−1/2, −*y*+1/2, *z*−1/2; (iv) −*x*, −*y*, −*z*+1; (v) *x*, −*y*, *z*−1/2; (vi) *x*−1/2, −*y*+1/2, *z*+1/2; (vii) −*x*+1/2, −*y*+1/2, −*z*; (viii) *x*, −*y*+1, *z*−1/2; (ix) −*x*, −*y*+1, −*z*+1; (x) −*x*+1, *y*, −*z*+1/2; (xi) *x*, *y*, *z*−1; (xii) *x*, *y*, *z*+1.

### Hydrogen-bond geometry (Å, º)

**Table:**

*D*—H···*A*	*D*—H	H···*A*	*D*···*A*	*D*—H···*A*
O*W*1—H11···O3^ii^	0.72 (3)	2.40 (4)	2.994 (4)	142 (5)
O*W*1—H11···O6^ii^	0.72 (3)	2.41 (3)	3.079 (4)	155 (5)
O*W*1—H12···O*H*	0.74 (3)	2.45 (3)	3.145 (4)	159 (4)
O*W*1—H12···O2	0.74 (3)	2.75 (4)	3.179 (4)	120 (4)
O*W*2—H21···O5	1.00 (4)	1.61 (4)	2.606 (3)	174 (4)
O*W*2—H22···O*W*3*B*^ii^	0.86 (4)	2.02 (4)	2.841 (9)	160 (4)
O*W*2—H22···O*W*3*A*^ii^	0.86 (4)	2.27 (4)	3.033 (10)	148 (4)
O*W*2—H22···O6^xiii^	0.86 (4)	2.57 (4)	2.973 (4)	109 (3)
O*H*—H1···O*W*1^ii^	0.69 (4)	2.22 (4)	2.891 (4)	165 (5)

Symmetry codes: (ii) −*x*+1/2, −*y*+1/2, −*z*+1; (xiii) *x*, −*y*, *z*+1/2.
